# Perspectives on Work in the Continuing Care Sector during and after the COVID-19 Pandemic: A Mixed-Method Design

**DOI:** 10.1155/2024/7187263

**Published:** 2024-04-18

**Authors:** Lindsay M. Guest, Janet McCabe, Chase O'Halloran, Maryam Rana, Winnie Sun, David Rudoler

**Affiliations:** ^1^Faculty of Health Sciences, Ontario Technology University, Oshawa, ON L1G 0C5, Canada; ^2^Ontario Shores Centre for Mental Health Sciences, Whitby, ON, Canada

## Abstract

**Background:**

Improving the recruitment and retention of healthcare workers in the continuing care sector is critical to ensuring adequate care for older adults, which was highlighted following the COVID-19 pandemic.

**Purpose:**

The purpose of this study was to understand the perceptions of prospective registered nurses about working in the continuing care sector and identify workplace attributes that attract prospective nurses to the sector.

**Methods:**

A sequential mixed methods study was conducted with nursing students at Ontario Tech University. Focus groups (*n* = 14) asked students to comment on views about working in the continuing care sector, and job attributes that may attract them to the sector. Focus group data were analyzed using thematic analysis. Subsequently, a cross-sectional survey asked students to respond to elicited choice job scenarios that varied job attributes. The job attributes were shaped by the focus group interview data. To assess respondent's preferences, the survey data (*n* = 139) were analyzed to generate willingness-to-pay (WTP) values for each job attribute.

**Results:**

Focus group interviews suggested that fair compensation, optimal client-to-staff ratios, unionized work environments, comprehensive benefits packages, and flexible work arrangements were important job attributes. In survey results, 18.0% expressed interest in working in the continuing care sector compared to 75.5% in acute care. Regression analysis suggested that higher amounts of paid vacation (WTP: −5.983; 95% CI: −13.749 and −0.037) and higher risk of injury (WTP: 0.684; 95% CI: 0.124 and 1.208) were associated with work in the continuing care sector. *Impact*. Continuing care workplaces can attract nurses by offering flexible options such as part-time positions and paid vacation and by taking actions that can mitigate the risk of workplace injury, violence, and abuse. Nursing students should be shown the positive aspects of working with older adults and dispel negative perceptions about the continuing care sector. Further research is needed to understand the preferences for work and risk perceptions among currently employed nursing staff.

## 1. Introduction

Globally, the recruitment and retention of healthcare workers in the continuing care sector is an increasingly significant challenge [1, 2]. Continuing care in Canada describes a complex system which includes delivery of all services provided by long-term care, home care, and home support (e.g., nursing homes, long-term care, and supportive living facilities), and combines aspects of both health and social services [3]. Typically, this is an undervalued workforce within Canadian society [4]. During the best of times, the continuing care sector has experienced difficulty recruiting and retaining healthcare workers [5–7]. It has been suggested that the sector suffers from a workforce crisis, where staffing levels are never high enough to meet demands [8]. Recruitment and retention challenges have been linked to workplace attributes, including staffing ratios as high as 1 worker to 40 clients, wages that are at market minimums, and precarious and part-time work [8–10].

Another important workplace attribute in the continuing care sector is workplace safety. A Canadian study found that more than 40% of institutional continuing care workers experience physical violence on a daily basis [11]. Understaffing, inappropriate resident placement, frequent and infrequent work, night shift work, lack of communication, and lack of collegial support have all been cited as causes of workplace violence [8, 11]. The perception that there are workplace risks, whether they exist or not, leads to challenges in recruitment for the continuing care sector, and the experience of safety risks on the job makes retaining staff difficult.

Workplace safety came to the fore during the COVID-19 pandemic, with the Canadian continuing care sector being hit particularly hard by the disease, with devastating effects for residents, families, and the workforce. According to early 2021 data, continuing care facility residents accounted for more than 60% of COVID-19-related deaths in Ontario [12]. Other resources have estimated that between 62% and 82% of deaths due to COVID-19 have been among residents of continuing care facilities, and 5 of 7 Ontario healthcare workers who have died from COVID-19 were personal support workers in the sector [4]. Low staff levels, part-time workers operating in multiple sites, lack of testing, and lack of personal protective equipment have been blamed for preventable COVID-19-related deaths in the sector [4]. These unfavourable working conditions contribute to job dissatisfaction in the continuing care sector, exacerbating the recruitment and retention crisis.

Nurses are critical healthcare providers who can best meet the growing demands imposed on the healthcare system by an aging population [13]. Unfortunately, clinical placements in continuing care facilities have frequently been unsatisfactory and/or unsettling for undergraduate nursing students, discouraging them from working in the continuing care sector in the future [14]. Understanding the perspectives of working in the continuing care sector is essential to ensure that nurses in this field have the necessary support and resources to provide quality care to their patients. Nurses face many challenges on the job, such as complex patient needs, increases in absenteeism, worsened mental health, overtime hours, and a high level of emotional labour [15, 16]. These challenges highlight the need for healthcare organizations to implement policies and practices that support the well-being of healthcare workers. This, in turn, can improve the quality of care delivered to patients.

Improving retention and recruitment efforts is critical to attracting new nurses to the field in order to service the increasing population over the age of 65 in need of healthcare services [15]. It is estimated that Canada will need to increase its healthcare workforce (across different sectors) by 80% by 2040 [17] to keep up with the current demand. The national turnover rates currently range from 8.8% to 37.0%, with variations depending on the nursing specialty and geographical location [18]. A high turnover rate in the nursing workforce can lead to lower quality of care, increased costs, and decreased patient satisfaction [18]. Increasing wages is one solution to recruiting and retaining a nursing workforce, but there may be other workplace attributes that can be utilized to attract and retain workers. These attributes include benefit packages, vacation time, staff-to-client ratios, safety, and more [4, 8–11]. Currently, there is limited research highlighting preferable work attributes within prospective nurses, especially in the Canadian continuing care sector, indicating a gap and need for research in this area.

The purpose of this study is to understand the perceptions that prospective registered nurses have about working in the continuing care sector and the workplace attributes that could attract prospective nurses to the sector. This study addresses the following research questions: (1) What are the major concerns of prospective nurses about working in the continuing care sector during and after the COVID-19 pandemic? and (2) What workplace attributes could attract prospective nurses to the continuing care sector?.

## 2. Materials and Methods

### 2.1. Design, Setting, and Participants

A sequential exploratory design was completed for this mixed-methods study [19]. The qualitative descriptive methodology was used first through focus group interviews, followed by quantitative survey methodology. The quantitative survey instrument was built based on the qualitative data that were previously collected during the focus group interviews. Participants were recruited using the convenience sampling method. Nursing students who were included in the study were enrolled at the University, where the research team was employed. The study interviewed nursing students to understand the perceptions they have about how to retain and recruit healthcare workers in the continuing care sector. Subsequently, an original survey instrument was used to elicit preferences for job attributes [20]. All persons who participated in the study were enrolled in the Bachelor of Science Nursing program at Ontario Tech University in Oshawa, Ontario. The four-year program is offered in collaboration with Durham College, and students have a practicum placement focused on the care of older adults in year 1 of their studies. The nursing program at Ontario Tech University is committed to offering students state-of-the-art learning environments, experience with leading-edge technology, and support praxis through clinical placements and simulation, positioning students for a successful career in healthcare and other sectors. The program had 840 enrolled students in the 2022/23 academic year.

This study was reviewed and approved by the Research Ethics Board at Ontario Tech University, Oshawa, ON, Canada (REB #16402).

### 2.2. Focus Groups: Data

A semistructured interview guide [21, 22] (see Supplementary Materials-Appendix A) was constructed by the research team for the focus groups. The focus group data collection was carried out between the University's fall semester of 2021 and the fall semester of 2022. Students were recruited via e-mail and asked to reach out to the research team to express interest in participating in the focus groups. One member of the research team (LG) hosted each focus group to maintain consistency. Interaction and discussion between participants were encouraged as much as possible. Focus groups were held virtually via Google Meet. Prior to each focus group, the researcher explained the purpose of the study, that participation was voluntary, the student's right to withdraw at any moment, that all data collected would be kept confidential, and that all identifiers would be removed. Before the online focus group began, each participant provided informed verbal consent. A demographic datasheet was completed by each attendee once informed consent was collected. The focus groups were audio and video recorded and transcribed verbatim. Transcripts were cleaned and all identifiers were removed prior to being shared with the remainder of the research team. Two members of the research team (DR and JM) reviewed the deidentified transcripts for familiarity prior to analysis. Data saturation was reached after 6 focus groups. Each focus group involved 2–4 students. The final focus group sample consisted of 14 participants.

### 2.3. Focus Groups: Data Analysis

Braun and Clarke's thematic analysis [23] was used to derive overarching themes from the focus group transcripts, highlighting relevant workplace attributes to inform the development of an elicited choice survey, a form of stated preference survey [24]. The data were carefully examined to identify patterns and latent level themes, which refer to underlying ideas not immediately apparent in the data. This process resulted in the development of a coding system. To ensure intercoder reliability [25], two researchers independently generated an initial code list in Microsoft Word and discussed it, reaching over 80% agreement [26]. The initially drafted codes were further developed into themes and subthemes after a rereading of the transcript data and subsequent discussions between DR and JM. A draft coding system was then provided to three other researchers (LG, CO, and MR) who applied the coding system to the transcript data, and through iterative team discussions, reached consensus on the coding system. The coding system was developed deductively and inductively. Our interview guide included specific workplace attributes derived from our review of the literature (e.g., compensation, workplace safety, vacation time, and benefits), and these same categories were incorporated into our initial codebook. However, given the lack of research on workplace preferences in the continuing care sector, we allowed for additional themes to be grounded in the raw data extracted from the focus group transcripts. Data analysis was conducted in English, the same language in which the focus groups were conducted.

### 2.4. Survey: Data

An online cross-sectional survey (see Supplementary Materials-Appendix B) was used to collect self-report data from students in the nursing program at Ontario Tech University during the fall 2022 semester. The survey was inspired by the work of Wiswall and Zafar, who used a survey instrument to study preferences for workplace attributes in a sample of undergraduate students at New York University [20]. Our survey consisted of the following five sections: a demographic section followed by four sections of hypothetical job scenarios. The demographic section collected data on year of study, age, gender, cumulative grade point average (GPA), plans postgraduation, whether they intend to work in the continuing care sector postgraduation, as well as postgraduation salary expectations. The remaining sections each contained eight job scenarios, with each scenario consisting of three job offers. The job offers differed by four attributes in each scenario. Participants were instructed that each attribute was identical across the job offers. We varied the job offers by the following attributes: hourly wage, percentage wage increases per year, vacation time, benefits, staff-to-patient ratio, patient acuity, hours worked per week, part-time option, unionized work environment, consistent shift hours, training and development opportunities, probability of being fired, and risk of workplace injury. Job attributes that were deemed most important to the participants in the focus group interviews were used in the construction of the job offers used in the survey. Other job attributes were inspired by those highlighted in the literature. Each of the offers was varied by four attributes at a time, and the values of the attributes (e.g., hourly wage) were designed to be as realistic as possible. We used new hire and union packages [27, 28] from local government-funded facilities and expert opinion from professionals in the continuing care sector in Ontario, Canada to develop realistic job attributes and variations in the attributes. Further details about how the job scenarios were constructed are provided in Supplementary Materials-Appendix C.

The survey allowed participants to provide elicited choice probabilities, which permitted respondents to show uncertainty about their preferences [24]. Participants were asked to rank each of the three hypothetical job offers in each scenario by assigning a percentage likelihood of choosing the scenario. Participants were informed that all values must sum to 100. Examples of these job scenarios are provided in Table 1. The survey data were extracted from the Ontario Tech University secured survey program (LimeSurvey).

### 2.5. Survey: Data Analysis

First, we estimated descriptive statistics for all variables in the demographic section of the survey. Following from Wiswall and Zafar [20], for our main analysis we estimated the log-odds of choosing each job as a function of the difference levels in job characteristics between job choices. To ensure the log-odds representation of our outcome variable, we made slight adjustments (adding or subtracting by 0.0001) to probabilities that were either 0 or 1. This was necessary because without these adjustments, the log transformation would not be valid. To account for the measurement error introduced when people round their choice probabilities to units of 5% or 10% during self-reporting of preference data, we utilized a least absolute deviations (LADs) estimator. Unlike the ordinary least squares estimator, which minimizes the squared differences between observed and predicted values, the LAD estimator minimizes the absolute differences. This approach increases the robustness of estimates by mitigating the impact of outliers and measurement errors [24].

We estimated a separate model for each participant who responded fully to each of the job scenarios. During the survey, participants were presented with 32 different scenarios and were asked to assign probabilities to three job options in each scenario. Since job 1 was used as the base case in each model, each participant had 32 × 2 = 96 unique observations. Job 1 was used as the reference case in each model, so 64 observations per participant were used in the regression model. We obtained coefficient estimates by averaging coefficients for each job attribute across participants. To perform inference, we used block bootstrap resampling, repeating the estimation process 5,000 times by randomly drawing participants (with replacement) from the sample. We then calculated the 95% bootstrap confidence intervals using the 2.5^th^ and 97.5^th^ percentiles of the resampling distribution. We also presented these results as individual-level willingness-to-pay (willingness-to-accept) statistics. All regression analyses were performed using R version 4.1.2. LAD estimates were generated by using the LAD function from the L1pack package [29].

## 3. Results

### 3.1. Focus Groups: Participant Characteristics

The focus group participants consisted of 14 Ontario Tech University nursing students; 3 (21.4%) male and 11 (78.6%) female (Table 2). Most focus group participants were under the age of 30 and represented a variety of ethnicities. Four participants had previous experience working in a continuing care setting.

### 3.2. Focus Groups: Data-Derived Themes and Codes

The findings from the focus groups revealed three important themes: (1) workplace attributes that support recruitment, (2) workplace attributes that support retention, and (3) perceived risks and fears related to work in the continuing care sector (Table 3).

A recurring theme among the focus group responses was *the work attributes of the workplace perceived to support recruitment* and thereby were enticing to the prospective nurses. Participants discussed attributes including adequate compensation, a preference for a unionized environment, the importance of comprehensive benefit packages, appropriate client-to-staff ratios, and work schedule flexibility. Client-to-staff ratios were raised several times by the participants, across all three themes in different ways. Students were concerned about the high patient-to-registered nurse ratios and the fact that high ratios increase the physical and emotional demands of nursing, limit one's ability to form personal relationships with clients, and may place nursing staff in situations which pose ethical dilemmas.

The second occurring theme among the focus group responses was the *work attributes that support retention*. These attributes reflect elements that are aimed at keeping the individuals employed over longer periods of time. Elements included a comprehensive orientation period which included information on roles, responsibilities, and expectations. Participants also highlighted staffing complements as a means of retention, with importance on the ratio and number of staff that form the care team, and the nature of the roles (personal support workers, registered practical nurses, and registered nurses). Additional elements cited were opportunities for continued growth and education along with skill development, the ability to form relationships with the clients, and working in an environment in which there were positive working relationships and support from both colleagues and management.

The final theme that emerged from the focus group responses was the *perceived risks and fears related to working in the continuing care sector*. Participant responses reflected both real and perceived risks and fears about working in the continuing care sector including the potential for abuse and violence towards the RN as well as towards the patients, and being engaged in situations that promoted or reinforced low standards of care which would lead to ethical and moral dilemmas. Furthermore, participants were concerned about the potential of knowledge and skill decay of acute care level skills if one was to spend a significant amount of time within the sector. The potential for burnout was a perceived risk, and participants spoke separately of the perceived fear of the physical and emotional toll of the work, including the potential for injury, and the nurse as martyr framework that pervades nursing as giving when you have nothing left to give, or giving and caring with little to no attention to one's own needs. The potential for understaffing and inadequate skills' mix of staff was a concern, along with a concern about the availability of the proper technology and equipment to properly function. Lastly, participants raised concerns about working in private or for-profit environments versus public care facilities, as many perceive public care facilities to be professionally enticing.

### 3.3. Survey: Participant Characteristics

Table 4 shows that 139 participants completed the demographics section of the survey. This represents approximately 20% of the four-year collaborative nursing program. The survey included 46 first-year students, 23 second-year students, 36 third-year students, and 34 students in their fourth or fifth year. Of the participants, 16 (11.5%) identified as male and 120 (86.3%) identified as female, gender nonconfirming, or other. Most participants were under 30 years old, which is typical for university-age students. Ontario Tech University uses a 4.3 GPA scale. The average cumulative GPA of the participants was 3.4 (SD = 0.451), equivalent to a B+. Out of the total participants, only 25 (18%) expressed interest in working in the continuing care sector after graduation. Most participants intended to seek full-time employment after graduation, with an average salary expectation of $65,654.14 (SD = $22,741.98).

### 3.4. Regression Analysis

Of the 139 participants in the survey, 67 fully responded to each of the job scenarios in the survey. We limited our regression analysis to the 88 participants who did not miss more than one job scenario in each of the four sections. In Appendix D, Table A1 in the Supplementary Materials, we compare the demographic characteristics of those included in the regression analysis with those who were excluded. Gender, choice of nursing sector, cumulative GPA, and salary expectations were similar across these groups. While none of the differences between the groups were statistically significant (*p* values >0.1), those who were included in the regression analysis tended to be older, in higher years of study, and more likely to report working full-time after graduation.

Table 5 shows our regression estimates. We interpret the sign of these estimates since the magnitudes are difficult to interpret. Thus, higher hourly wages, wage increases per year, part-time options, unionized environment, vacation time, consistent shifts, and training and development were positive predictors of the hypothetical job preference. Meanwhile, hours worked, probability of being laid off, staff-to-client ratio, risk of injury, and patient acuity were negative predictors of hypothetical job preference.

Table 6 shows the willingness-to-pay (WTP) estimates. These can be interpreted as the increase in hourly wages needed for prospective nurses to accept an undesirable workplace attribute, or wages nurses are willing to forgo for a desirable attribute. Only vacation and risk of injury had statistically significant WTP estimates. On average, respondents were willing to forgo $5.98 in hourly wages for a one-week per year increase in paid vacation time. While this estimate suggests respondents highly valued paid vacation, our data were consistent with a wide range of WTP values (95% CI: −13.749 and −0.037). On average, respondents needed to receive an increase of $0.68 in hourly wages to accept a 1% increase in the risk of injury (95% CI: 0.124 and 1.208).

## 4. Discussion

This study aimed to explore the views of aspiring registered nurses regarding employment in the continuing care sector. Focus group interviews conducted with nursing students revealed key job attributes for recruitment, including fair compensation, optimal client-to-staff ratios, unionized work environments, comprehensive benefit packages, and flexible work schedule arrangements. Client-to-staff ratios were brought up several times during the interviews, and students highlighted the effect these have on nurses' ability to provide safe and compassionate care. Participants were also concerned about the real and perceived risks of working in the continuing care sector, including the potential for abuse and violence toward healthcare workers and patients. There was also an expressed concern about the skill and knowledge decay, particularly while working long-term in the continuing care sector.

These perspectives on workplace attributes informed the development of a survey of nursing students at Ontario Tech University, particularly the hypothetical job scenarios. Although there was considerable variation in the individual preferences expressed in the survey data, two attributes more consistently stood out as important to prospective nurses: paid vacation and risk of injury. The preference for jobs with longer periods of paid vacation aligns with the emphasis on workplace flexibility and work-life balance that arose during the focus group discussions. Similarly, the preference for jobs with lower risk of injury reflects the concerns expressed about safety in the continuing care sector.

Few previous studies have explored the preferences for workplace attributes in the continuing care sector or the views of prospective nurses. One study conducted in South Korea surveyed recently graduated nurses on the importance of different workplace attributes. They found that salary, working conditions, and organizational climate were the most important factors when choosing a workplace [30]. Studies conducted in other countries (such as Australia, South Korea, the United States, the United Kingdom, China, and France [13, 31–39]) have focused on the perspectives of nurses already working in their continuing care sectors and the factors that contributed to their decisions to leave the sector. These studies highlighted job security [31, 32], workplace safety [33, 34], favourable work hours [13, 31, 35–37], paid vacation [36, 37], recognition of work [38], and sick leave [32, 34, 39] as important workplace attributes. These studies show some overlap with the factors identified in our data, but other factors may have emerged given differences in context and differences in career stages. Further research in the Canadian context on the views of practicing and later career nurses is needed to understand how preferences for workplace attributes may evolve later in their careers.

Our focus group and survey data also indicate a lack of enthusiasm for working in the continuing care sector. Specifically, we found that only 18% of survey respondents expressed interest in working in this sector after graduation. This is striking in contrast with the 76% who showed interest in working in acute care. Previous research has identified a similar apprehension. For example, the study conducted in Australia noted that only 8% of undergraduate nursing students chose to work with older adults, and this preference further declined throughout their studies [40]. Another survey of nursing students conducted in the United States found that while attitudes toward working with older adults improved over the duration of their studies, working with this population consistently ranked last for workplace preferences [41].

The results emphasize that ageist views and stigma towards the continuing care sector are prevalent within an undergraduate nursing population. Nursing education programs (both at the Bachelor level and Diploma level) need to work to actively to address these views to improve the recruitment of healthcare professionals into the continuing care sector. Several solutions to these problems have been suggested in the literature, including curricula reviews to ensure that there is a focus on the needs of older adults, access to role models who can positively promote work in the sector [42], practicum experiences in the continuing care sector [41], and integrating gerontology focused simulations into nursing programs [43]. Focus on spreading out practicum experiences throughout the duration of a degree or diploma would be important as this could assist students in connecting continuing care with early-level learning and increase student's understanding of the complexity of continuing care. Concerns about skill decay in the continuing care sector may also be reinforced by the curricula that focus on the technical aspects of nursing, which may direct prospective nurses to the acute care sector [42]. Overall programs could focus on student attitudes toward older adults as well as highlighting the skills required of those nurses working in the sector, and the opportunities that are available (e.g., gerontology certificates and the use of technical skills specific to continuing care).

More research is needed on multilayered educational interventions to better understand how to support students who enter the continuing care sector after graduation. It is important to go beyond just studying student attitudes toward elderly populations, as it is unclear if attitude alone is the main factor influencing work location.

Workplaces within the continuing care sector also have a role in addressing the perception of working in the sector. The creation and promotion of work environments that allow staff to exercise their full scope of practice (for registered nurses and registered practical nurses) would allow for both caring relationships and the use of technical skills. Furthermore, workplaces that provide patient-to-nurse ratios that allow for the development of caring relationships may ease negative perceptions concerning workload. High registered nurse-to-patient ratios also limit the opportunity to work to one's full scope of practice and may limit the roles and responsibilities that registered nurses take on within the sector. Educational organizations can play their part by highlighting the potential for practicing to one's full scope of practice during practicum experiences.

Workplaces in the continuing care sector can attract prospective nurses by providing flexible work options, including part-time positions and paid vacation. In addition, it is important for these workplaces to take measures to reduce the actual or perceived risks of workplace injury, violence, and abuse. This is especially important after the COVID-19 pandemic, which may have exacerbated perceived risks. Some evidence suggests that risk-averse nurses tend to overestimate the risks associated with their job [44]. Further research is necessary to understand the risk perceptions of both prospective and currently employed nursing staff [45]. Currently, there is a lack of studies examining interventions aimed at addressing workplace risk perceptions.

### 4.1. Limitations and Strengths

In the study, there were several limitations that should be taken into consideration. First, the focus group sample size included 14 participants interviewed during six different focus group meetings. Despite the small focus group sample size, the aim of our qualitative descriptive study is to achieve transferability of study findings with the goal of providing an initial understanding of the phenomena of interest and their implications. Second, our survey was not implemented with a randomized sample of nursing students. While randomization helps to minimize bias and increase the generalizability of the results, it was not feasible in our study environment. We have made efforts to highlight the characteristics of our sample to aid in assessing external validity. Third, 51 survey respondents who did not complete the job attributes section were excluded from the main quantitative results. These nonrespondents were mostly younger students in earlier years of study. The views of third-year and fourth-year students may be more relevant for understanding the perspectives of prospective nurses, as they are closer to entering the job market. However, our findings may be less applicable to students in the earlier stages of their studies. Fourth, it is important to acknowledge that the study focused exclusively on nursing students. While this allowed for a specific examination of this group, it also means that the findings may not be applicable to other healthcare professionals or currently employed nurses. More research is required to investigate the experiences and perspectives of these additional groups.

Our study had several notable strengths. First, we successfully integrated qualitative results into the design of the quantitative survey, creating an integrated mixed methods study. Second, we used hypothetical job scenarios to eliminate biases that arise when analyzing actual job choices made by workers. Realized job choices can be influenced by employer preferences and job availability, rather than true worker preferences [20]. Third, this study aimed to pilot an approach for understanding healthcare professional preferences regarding workplace attributes in Canada. It could serve as the initial step towards gaining a deeper understanding of how continuing care workplaces can attract healthcare professionals. Despite being an initial step, this study should still provide valuable insights to employers in the continuing care sector.

## 5. Conclusions

Cross-sector wage competitiveness remains crucial for the continuing care sector to attract and retain healthcare professionals. However, this study emphasizes additional workplace attributes that employers in the sector should consider when recruiting and retaining prospective nurses. Employers should prioritize creating a flexible work environment that allows healthcare professionals, including registered nurses, to work to their full scope of practice. In addition, workplaces need to address the real and perceived risks of injury, abuse, and violence that influence labour market decisions. Nursing curricula and postsecondary faculty engagement in nursing education play a key role in addressing negative perceptions about the sector, which may be unintentionally reinforced by the current curricula (for example, relegating continuing care sector placement to initial practicum experiences). Nursing faculty should consider opportunities that explore the complexity of work in the long-term care sector. These may include simulations focused on providing care to dementia patients [46], structured field experiences across programs with long-term care homes [47], and case studies that raise the nuanced and professional-based issues that are reflected in this paper (e.g., advocating for care standards and negotiating opportunities to work to full scope). Nursing education research is required in this area to demonstrate the effectiveness of these types of initiatives on student attitudes toward the sector. These efforts may expose nursing students to the positive aspects of working within the continuing care sector and with older adults while simultaneously working to dispel negative perceptions about the continuing care sector.

## Figures and Tables

**Table 1:** Example choice scenarios.

Section A	Hourly wage	Annual percentage increase in earnings	Average work hours per week for full-time	Work flexibility: is part-time available?
*Example*				
Job 1	$35.36	2%	35	Yes
Job 2	$35.94	2.5%	40	No
Job 3	$34.00	3%	44	Yes

Section B	Hourly wage	Probability of you being fired or laid off from the job in the next year	Union vs. nonunion environment	Staff-to-patient ratios

*Example*				
Job 1	$38.97	1%	Union	1 : 61
Job 2	$38.33	3%	Nonunion	1 : 90
Job 3	$38.48	7%	Union	1 : 56

Section C	Hourly wage	Amount of vacation of paid time off	Shift work/rotation/preferable hours	Opportunity for training and development

*Example*				
Job 1	$36.54	4 weeks	Consistent shifts	$2,208
Job 2	$39.00	3 weeks	Rotation shifts	$3,996
Job 3	$40.93	2 weeks	Consistent shifts	$3,956

Section D	Hourly wage	Available benefits and pension package	Risk of injury on the job	Patient acuity

*Example*				
Job 1	$36.84	13%	6.2	Stable
Job 2	$36.67	10%	13.7	Moderate
Job 3	$40.63	9%	24.8	Complex

**Table 2:** Focus group demographics.

Variable	Frequency	Percent (%)
*Year of study*		
1^st^ year	7	50
2^nd^ year	2	14.3
3^rd^ year	1	7.1
4^th^ year	4	28.6
*Age range*		
18–29	9	64.3
30–39	1	7.1
40–49	4	28.6
*Gender*		
Male	3	21.4
Female	11	78.6
*Ethnicity*		
Caucasian	3	21.4
African-American	4	28.6
Asian	3	21.4
Two ethnicities or more	2	14.3
Other	2	14.3
*Highest education*		
High school diploma	5	35.7
College	3	21.4
Bachelor's degree	5	35.7
Master's degree	1	7.1
Continuing care placement		
Yes	2	14.3
No	10	71.4
No, but employed in LTC	2	14.3

**Table 3:** Focus group's data-derived themes, codes, and selective illustrative quotes.

Theme	Code	Illustrative quote
Workplace attributes that support recruitment	Adequate compensation	“Wage compensation is important for me”
	Unionized environment	“Unionized environment is very important”
	Benefits	“I would like to work full-time when I get a job because of benefits”
	Client-to-staff ratio	“We don't have enough time because there just two of us to take care of 15 people in the morning, we have to get six people up. You don't have enough time to do much”
	Work flexibility (personal choice)	“You need to know what you're going to be working on and it needs to be predictable set of shifts”

Workplace attributes that support retention	Orientation	“I'm told what my job expectations are and if I have someone to guide me as to how to do my duties and teach me, those are things I look for in an orientations that can prepare me for success in my job”
	Staffing complement	“I'll say the issues with staffing, for example, if they have adequate staff so that there's no burnout. I work in a long-term care, we get burnt out. We work so many hours because people don't show up for work. So I'd like a workplace that has adequate staff”
	Continued education, training, and skill development	“I'm looking for a place to build a career in. So for me I would like opportunities for advancement, you know if I wanted to take a specialty or move up then I want to be able to stay within the same organization”
	Personal relationships with clients	“So in my experience, it's just the relationships that you get to make with residents again because you're seeing the same people, you know, daily or weekly”
	Reliable and supportive coworkers/management	“So I agree with everybody that the main thing is respect for the employee, so respect for me as an individual, but also a group. If I feel like management cares about me, then I care about the job more”

Perceived risks and fears related to work in the continuing care sector	Abuse and violence	“A risk of injury and verbally, emotionally, and trauma, I think that is very important to have security in place. You want more to minimize any kind of injury, like physically, verbally, and emotionally”
	Ethical and moral dilemmas	“It doesn't allow you to actually provide adequate care for a patient because, for one thing, in the back of your mind you're concerned about getting all your meds done, but you're not actually getting to analyze”
	Knowledge and skill decay	“It's really easy to forget, really simple things”
	Burnout	“The workload in the nursing home is too much”
	Understaffing and poor skill mix	“We don't have enough time because they're just two of us to take care of 15 people in the morning, we have to get six people up. You don't have enough time to do much”
	Physically and emotionally demanding work	“I would be most concerned about the risk of injury. I think I would try to have a job that is on the lower side of risk of injury”
		“Because sometimes you have to think a little selfish and think of things that benefit you and if you have a family, that can benefit your family, take vacations, have time for yourself to just relax, away from work”
	Working in public vs. private long-term care homes	“But like as soon as it came down to making a profit, a lot of standards went out the window or they try to get away with like sweeping it out of the door, really quietly”
	Availability of resources	“My previous placements, when I asked where the suction is, and where the crash cart is they're like “oh, we don't have one””
		“…If you see someone who's had a stroke and they clearly need way more care than they're getting, it's difficult to not be able to do anything”

**Table 4:** Survey demographics.

Variable	All
Number of participants	139
Gender	
Male	16 (11.5%)
Female, gender nonconforming, and other^1^	120 (86.3%)
Prefer not to answer	3 (2.2%)
Year of study	
1st year	46 (33.1%)
2nd year	23 (16.5%)
3rd year	36 (25.9%)
4th and 5th year^2^	34 (24.4%)
Age group	
18–21	72 (51.8%)
22–29	34 (24.5%)
30–39	15 (10.8%)
40+	18 (12.9%)
Cumulative GPA^3^	3.4 (0.451)^4^
Future nursing sector^5^	
Home community care	36 (25.9%)
Continuing/long-term care	25 (18.0%)
Primary care	59 (42.4%)
Acute care	105 (75.5%)
Do not plan on working in healthcare	1 (0.7%)
After graduation plans	
Work Full-time	103 (74.1%)
Work Part-time	9 (6.5%)
Other^6^	27 (19.4%)
Salary expectations^7^	$65,654.14 (22,741.98)^4^

^1^Combined due to small cell sizes. ^2^One respondent in 5^th^ year. ^3^Based on 125 observations. ^4^Std. dev. ^5^Based on 130 observations. ^6^Combines the following categories: attend graduate or professional school, take time off, and other. ^7^Based on 133 observations.

**Table 5:** Survey regression analysis results.

Number of participants	*n* = 88		
Variable	Estimate	Std dev.	95% CI

Hourly wages	0.241	0.038	(0.168, 0.319)
Wage increase per year	0.451	0.113	(0.226, 0.671)
Hours worked	−0.092	0.019	(−0.13, −0.057)
Part-time option	0.763	0.237	(0.319, 1.265)
Probability of being laid off	−0.185	0.051	(−0.291, −0.089)
Unionized environment	1.291	0.205	(0.909, 1.706)
Staff-to-client ratio	−0.053	0.008	(−0.069, −0.039)
Vacation	0.531	0.143	(0.254, 0.813)
Consistent shifts	1.394	0.343	(0.707, 2.074)
Training and development	0.035	0.013	(0.009, 0.061)
Benefits	0.075	0.078	(−0.074, 0.230)
Risk of injury	−0.137	0.019	(−0.176, −0.100)
Patient acuity	−0.481	0.094	(−0.679, −0.306)

**Table 6:** Survey willingness-to-pay (WTP) regression analysis results.

Number of participants	*n* = 88		
Variable	WTP	Std dev.	95% CI

Wage increase per year	−1.378	1.156	(−3.573, 0.951)
Hours worked	0.142	0.378	(−0.622, 0.856)
Part-time option	−0.811	6.466	(−12.665, 13.093)
Probability of being laid off	−0.262	1.620	(−3.901, 2.346)
Unionized environment	−8.631	9.852	(−29.066, 10.171)
Staff-to-client ratio	0.341	0.325	(−0.17, 1.09)
Vacation	−5.983	3.425	(−13.749, −0.037)
Consistent shifts	−8.451	9.418	(−25.532, 11.284)
Training and development	−0.493	0.507	(−1.613, 0.365)
Benefits	−0.090	0.751	(−1.534, 1.403)
Risk of injury	0.684	0.280	(0.124, 1.208)
Patient acuity	0.910	5.150	(−10.383, 10.106)

## Data Availability

The qualitative and quantitative data used to support the findings of this study are available from the corresponding author upon request.

## Supplementary Materials

Supplementary Materials The Supplementary Materials that are referenced in the study include: Appendix A: semistructured interview-focus group guide. Appendix B: online cross-sectional survey instrument. Appendix C: job attribute justifications. Appendix D: Table A1 results where the researcher (DR) compared the demographic characteristics of those included in the regression analysis to those who were excluded.
