# Supplementary material for: Perspectives on Work in the Continuing Care Sector during and after the COVID-19 Pandemic: A Mixed-Method Design
Source: J Nurs Manag. 2024 Apr 18;2024:7187263. doi: 10.1155/2024/7187263 (PMC11919170; doi:10.1155/2024/7187263)
Supplement: Supplementary Materials — The Supplementary Materials that are referenced in the study include: Appendix A: semistructured interview-focus group guide. Appendix B: online cross-sectional survey instrument. Appendix C: job attribute justifications. Appendix D: Table A1 results where the researcher (DR) compared the demographic characteristics of those included in the regression analysis to those who were excluded. [file 7187263.f1.zip › Appendix A - Focus Group Guide.docx]

Focus Group Guide

1. Read through, answer questions and complete consent form for each participant
2. Demographic Form

|  | Year of study | Gender |
| --- | --- | --- |
| Participant #1 |  |  |
| Participant #2 |  |  |
| Participant #3 |  |  |
| Participant #4 |  |  |
| Participant #5 |  |  |
| Participant #6 |  |  |
| Participant #7 |  |  |
| Participant #8 |  |  |

1. What are you looking for in a job opportunity regardless of location?
2. What type of orientation is important to help you feel ready for the new environment?
3. What professional development do you feel would be important as you take on a new role?
4. What sector do you see yourselves working in after graduation?
5. What are your thoughts on a job in LTC?
   1. What do you like/dislike?
   2. What would deter you from LTC?
   3. What is your perception of nursing care that is provided in LTC facilities?
   4. What do you think your day-to-day job would look like?
   5. How have previous rewarding experiences in placements formed your opinions in relation to working in LTC?
6. What aspects about each of the following are important to you?

- Wage compensation
- Benefits
- Risk of injury (physical, verbal and psychosocial trauma)
- Opportunity for training and development
- Vacation
- Job security
- Staffing models (who is on the care team, ratios of RN’s to RPN’s to PSW’s, on-site physicians, on-site NP’s, patient to RPN ratio)
- Part time/Full time
- Shift work - preferable hours (evening, morning, afternoon)
- Patient acuity - how sick are they?
- Unionized environment
- Working with unregulated health providers
