# Supplementary material for: Perspectives on Work in the Continuing Care Sector during and after the COVID-19 Pandemic: A Mixed-Method Design
Source: J Nurs Manag. 2024 Apr 18;2024:7187263. doi: 10.1155/2024/7187263 (PMC11919170; doi:10.1155/2024/7187263)
Supplement: Supplementary Materials — The Supplementary Materials that are referenced in the study include: Appendix A: semistructured interview-focus group guide. Appendix B: online cross-sectional survey instrument. Appendix C: job attribute justifications. Appendix D: Table A1 results where the researcher (DR) compared the demographic characteristics of those included in the regression analysis to those who were excluded. [file 7187263.f1.zip › Appendix B - Survey.docx]

# Perspectives on work in the continuing care sector during and after the COVID-19 pandemic: Survey for Ontario Tech Nursing students

## Demographics

1. Year of Study of the BScN Program at Ontario Tech
   1. First
   2. Second
   3. Third
   4. Fourth
2. Gender
   1. Male
   2. Female
   3. Other
3. Age Group
   1. 18-21
   2. 22-29
   3. 30-39
   4. 40-49
   5. 50+
4. What is your current GPA (rounded to the nearest decimal point – e.g., 3.8)?
5. Do you see yourself working in the nursing sector at some point in the future?
   1. Yes
   2. No
   3. Not sure
6. What sector of the healthcare system do you see yourself working in upon graduation?
   1. Home and Community Care
   2. Long-Term Care
   3. Primary Care
   4. Acute Care
   5. Other
7. What are you planning to do after graduation?
   1. Work full-time
   2. Work part-time
   3. Attend graduate/professional school
   4. Take some time off
   5. I don’t know yet
   6. Other
8. What will be your annual starting salary in your first job post-graduation (please omit $ from your response)?

## Job Offer Scenarios: Section A

In each of the scenarios in this section, you will be shown hypothetical job offers. Each job offer is characterized by:

- Hourly wage earnings working full-time
- Annual % increase in earnings
- Average work hours per week for full-time
- Work Flexibility: is part-time work available?

These jobs are otherwise identical in all other aspects.

When answering these questions, envision that you have completed your program, graduated and are seeking employment when you are 30 years of age or older with experience. You have been offered each of these jobs, and now you have to decide which one to choose.

In each scenario, you will be asked for the percent chance (or chances out of 100) of the likelihood of you choosing each of the alternatives. State each chance as a number between 0 and 100 and the chances given to the three alternatives should **add up to 100**.

- 2-5% would mean “almost no chance”
- ~19% would be along the lines of “not much chance”
- 47-55% would mean “pretty even chance”
- ~82% would indicate there to be “a very good chance”
- 95-98% would mean “almost certain”

For example, if you give 3% chance to one option, it means that there is a very low possibility that you would choose that job scenario. On the other hand, if you give an 88% chance to a job scenario, it means that it is quite likely that you will choose it.

The percent chance can also be thought of as the number of chances out of 100.

Here is an example. Imagine that you are playing baseball. Say, when asked about their percent chance that your team will win, you answer 70. This means that you believe your team would win 70 out of 100 games on average if you played a number of games.

In the remainder of the survey, when entering your answers to questions that ask you about the percent chance of something, please do not use a percent sign, i.e., %.

1. You have been offered the following 3 jobs when you are aged 30 or older.

|  | (a) | (b) | (c) | (d) |
| --- | --- | --- | --- | --- |
|  | Earnings per year working full-time | Annual % increase in earnings | Average work hours per week for full-time | Work flexibility: is part time available? |
| Job 1 | $35.36 | 2% | 35 | Yes |
| Job 2 | $35.94 | 2.5% | 40 | No |
| Job 3 | $34.00 | 3% | 44 | Yes |

For example, if you were to choose Job 1, (a) your annual earnings would be **$35.36** if working full time; (b) your earnings would increase **2%** per year onwards until retirement, (c) you would have to work **35**  hours per week on average, and, (d) you **do** have the option of working part-time (part-time is defined as working at most half as many hours as full-time work and for half of the full-time salary).

**These jobs are otherwise identical in all other aspects.**

Now consider the situation where you are given the jobs offered above when you are aged 30, and you have decided to accept one of these jobs. What is the percent (%) chance (or chances out of 100) that you will choose each of these jobs?

The chance of each job should be a number between 0 and 100 and the chances given to the three jobs should add up to 100.

**Percent Chance**

Job 1 _____________

Job 2 _____________

Job 3 _____________

1. You have been offered the following 3 jobs when you are aged 30.

|  | (a) | (b) | (c) | (d) |
| --- | --- | --- | --- | --- |
|  | Earnings per year working full-time | Annual % increase in earnings | Average work hours per week for full-time | Work flexibility: is part time available? |
| Job 1 | $40.48 | 3% | 37.5 | No |
| Job 2 | $41.65 | 2% | 40 | Yes |
| Job 3 | $35.88 | 4% | 35 | No |

For example, if you were to choose Job 1, (a) your annual earnings would be **$40.48** if working full time; (b) your earnings would increase **3%** per year onwards until retirement, (c) you would have to work **37.5** hours per week on average, and, (d) you **do not** have the option of working part-time (part-time is defined as working at most half as many hours as full-time work and for half of the full-time salary).

**These jobs are otherwise identical in all other aspects.**

Now consider the situation where you are given the jobs offered above when you are aged 30, and you have decided to accept one of these jobs. What is the percent (%) chance (or chances out of 100) that you will choose each of these jobs?

The chance of each job should be a number between 0 and 100 and the chances given to the three jobs should add up to 100.

**Percent Chance**

Job 1 _____________

Job 2 _____________

Job 3 _____________

1. You have been offered the following 3 jobs when you are aged 30.

|  | (a) | (b) | (c) | (d) |
| --- | --- | --- | --- | --- |
|  | Earnings per year working full-time | Annual % increase in earnings | Average work hours per week for full-time | Work flexibility: is part time available? |
| Job 1 | $40.56 | 2.5% | 44 | Yes |
| Job 2 | $36.09 | 5% | 40 | No |
| Job 3 | $40.41 | 3% | 37.5 | Yes |

For example, if you were to choose Job 1, (a) your annual earnings would be **$40.56** if working full time; (b) your earnings would increase **2.5%** per year onwards until retirement, (c) you would have to work **44** hours per week on average, and, (d) you **do** have the option of working part-time (part-time is defined as working at most half as many hours as full-time work and for half of the full-time salary).

**These jobs are otherwise identical in all other aspects.**

Now consider the situation where you are given the jobs offered above when you are aged 30, and you have decided to accept one of these jobs. What is the percent (%) chance (or chances out of 100) that you will choose each of these jobs?

The chance of each job should be a number between 0 and 100 and the chances given to the three jobs should add up to 100.

**Percent Chance**

Job 1 _____________

Job 2 _____________

Job 3 _____________

1. You have been offered the following 3 jobs when you are aged 30.

|  | (a) | (b) | (c) | (d) |
| --- | --- | --- | --- | --- |
|  | Earnings per year working full-time | Annual % increase in earnings | Average work hours per week for full-time | Work flexibility: is part time available? |
| Job 1 | $33.75 | 4% | 44 | No |
| Job 2 | $41.30 | 2% | 50 | Yes |
| Job 3 | $38.67 | 3% | 40 | No |

For example, if you were to choose Job 1, (a) your annual earnings would be **$33.75** if working full time; (b) your earnings would increase **4%** per year onwards until retirement, (c) you would have to work **44** hours per week on average, and, (d) you **do not** have the option of working part-time (part-time is defined as working at most half as many hours as full-time work and for half of the full-time salary).

**These jobs are otherwise identical in all other aspects.**

Now consider the situation where you are given the jobs offered above when you are aged 30, and you have decided to accept one of these jobs. What is the percent (%) chance (or chances out of 100) that you will choose each of these jobs?

The chance of each job should be a number between 0 and 100 and the chances given to the three jobs should add up to 100.

**Percent Chance**

Job 1 _____________

Job 2 _____________

Job 3 _____________

1. You have been offered the following 3 jobs when you are aged 30.

|  | (a) | (b) | (c) | (d) |
| --- | --- | --- | --- | --- |
|  | Earnings per year working full-time | Annual % increase in earnings | Average work hours per week for full-time | Work flexibility: is part time available? |
| Job 1 | $38.10 | 3% | 48 | Yes |
| Job 2 | $34.17 | 4.5% | 35 | No |
| Job 3 | $40.49 | 2.5% | 40 | Yes |

For example, if you were to choose Job 1, (a) your annual earnings would be **$38.10** if working full time; (b) your earnings would increase **3%** per year onwards until retirement, (c) you would have to work **48** hours per week on average, and, (d) you **do** have the option of working part-time (part-time is defined as working at most half as many hours as full-time work and for half of the full-time salary).

**These jobs are otherwise identical in all other aspects.**

Now consider the situation where you are given the jobs offered above when you are aged 30, and you have decided to accept one of these jobs. What is the percent (%) chance (or chances out of 100) that you will choose each of these jobs?

The chance of each job should be a number between 0 and 100 and the chances given to the three jobs should add up to 100.

**Percent Chance**

Job 1 _____________

Job 2 _____________

Job 3 _____________

1. You have been offered the following 3 jobs when you are aged 30.

|  | (a) | (b) | (c) | (d) |
| --- | --- | --- | --- | --- |
|  | Earnings per year working full-time | Annual % increase in earnings | Average work hours per week for full-time | Work flexibility: is part time available? |
| Job 1 | $35.31 | 2% | 37.5 | No |
| Job 2 | $33.88 | 4% | 44 | Yes |
| Job 3 | $35.00 | 3% | 48 | No |

For example, if you were to choose Job 1, (a) your annual earnings would be **$35.31** if working full time; (b) your earnings would increase **2%** per year onwards until retirement, (c) you would have to work **37.5** hours per week on average, and, (d) you **do not** have the option of working part-time (part-time is defined as working at most half as many hours as full-time work and for half of the full-time salary).

**These jobs are otherwise identical in all other aspects.**

Now consider the situation where you are given the jobs offered above when you are aged 30, and you have decided to accept one of these jobs. What is the percent (%) chance (or chances out of 100) that you will choose each of these jobs?

The chance of each job should be a number between 0 and 100 and the chances given to the three jobs should add up to 100.

**Percent Chance**

Job 1 _____________

Job 2 _____________

Job 3 _____________

1. You have been offered the following 3 jobs when you are aged 30.

|  | (a) | (b) | (c) | (d) |
| --- | --- | --- | --- | --- |
|  | Earnings per year working full-time | Annual % increase in earnings | Average work hours per week for full-time | Work flexibility: is part time available? |
| Job 1 | $39.92 | 2% | 35 | Yes |
| Job 2 | $35.41 | 5% | 44 | No |
| Job 3 | $38.17 | 3% | 40 | Yes |

For example, if you were to choose Job 1, (a) your annual earnings would be **$39.92** if working full time; (b) your earnings would increase **2%** per year onwards until retirement, (c) you would have to work **35** hours per week on average, and, (d) you **do** have the option of working part-time (part-time is defined as working at most half as many hours as full-time work and for half of the full-time salary).

**These jobs are otherwise identical in all other aspects.**

Now consider the situation where you are given the jobs offered above when you are aged 30, and you have decided to accept one of these jobs. What is the percent (%) chance (or chances out of 100) that you will choose each of these jobs?

The chance of each job should be a number between 0 and 100 and the chances given to the three jobs should add up to 100.

**Percent Chance**

Job 1 _____________

Job 2 _____________

Job 3 _____________

1. You have been offered the following 3 jobs when you are aged 30.

|  | (a) | (b) | (c) | (d) |
| --- | --- | --- | --- | --- |
|  | Earnings per year working full-time | Annual % increase in earnings | Average work hours per week for full-time | Work flexibility: is part time available? |
| Job 1 | $34.05 | 3% | 40 | No |
| Job 2 | $34.06 | 2.5% | 44 | Yes |
| Job 3 | $36.00 | 2% | 50 | No |

For example, if you were to choose Job 1, (a) your annual earnings would be **$34.05** if working full time; (b) your earnings would increase **3%** per year onwards until retirement, (c) you would have to work **40** hours per week on average, and, (d) you **do not** have the option of working part-time (part-time is defined as working at most half as many hours as full-time work and for half of the full-time salary).

**These jobs are otherwise identical in all other aspects.**

Now consider the situation where you are given the jobs offered above when you are aged 30, and you have decided to accept one of these jobs. What is the percent (%) chance (or chances out of 100) that you will choose each of these jobs?

The chance of each job should be a number between 0 and 100 and the chances given to the three jobs should add up to 100.

**Percent Chance**

Job 1 _____________

Job 2 _____________

Job 3 _____________

## Job Offer Scenarios: Section B

In each of the scenarios in this section, you will be shown hypothetical job offers. Each job offer is characterized by:

- Hourly wage earnings working full-time
- Probability of being laid off over a one-year period
- Union vs Non-Union Environment
- Staff-to-patient ratios

These jobs are otherwise identical in all other aspects.

When answering these questions, envision that you have completed your program, graduated and are seeking employment in the nursing field. You have been offered each of these jobs, and now you have to decide which one to choose.

In each scenario, you will be asked for the percent chance (or chances out of 100) of the likelihood of you choosing each of the alternatives. State each chance as a number between 0 and 100 and the chances given to the three alternatives should **add up to 100**.

- 2-5% would mean “almost no chance”
- ~19% would be along the lines of “not much chance”
- 47-55% would mean “pretty even chance”
- ~82% would indicate there to be “a very good chance”
- 95-98% would mean “almost certain”

For example, if you give 3% chance to one option, it means that there is a very low possibility that you would choose that job scenario. On the other hand, if you give an 88% chance to a job scenario, it means that it is quite likely that you will choose it.

1. You have been offered the following 3 jobs when you are aged 30.

|  | (a) | (b) | (c) | (d) |
| --- | --- | --- | --- | --- |
|  | Earnings per year working full-time | Probability of you being fired or laid off from the job in the next year | Union vs Non-Union Environment | Staff-to-patient ratios |
| Job 1 | $38.97 | 1% | Union | 1:61 |
| Job 2 | $38.33 | 3% | Non-Union | 1:90 |
| Job 3 | $38.48 | 7% | Union | 1:56 |

For example, if you were to choose Job 1, (a) your annual earnings would be **$38.97** if working full time; (b) the likelihood of being fired from the job over the next year would be **1%**, (c) depending on your relative performance (i.e., how your work performance compares to that of your peers), you would be working in a **Unionized** environment; and, (d) there would be approximately a **1:61** staff to patient ratio.

**These jobs are otherwise identical in all other aspects.**

Now consider the situation where you are given the jobs offered above when you are aged 30, and you have decided to accept one of these jobs. What is the percent (%) chance (or chances out of 100) that you will choose each of these jobs?

The chance of each job should be a number between 0 and 100 and the chances given to the three jobs should add up to 100.

**Percent Chance**

Job 1 _____________

Job 2 _____________

Job 3 _____________

1. You have been offered the following 3 jobs when you are aged 30.

|  | (a) | (b) | (c) | (d) |
| --- | --- | --- | --- | --- |
|  | Earnings per year working full-time | Probability of you being fired or laid off from the job in the next year | Amount of bonus based on relative performance (% of income) | Proportion of men in the firm in similar positions |
| Job 1 | $39.52 | 5% | Non-Union | 1:84 |
| Job 2 | $40.62 | 6% | Union | 1:114 |
| Job 3 | $39.05 | 2% | Non-Union | 1:69 |

For example, if you were to choose Job 1, (a) your annual earnings would be **$39.52** if working full time; (b) the likelihood of being fired from the job over the next year would be **5%**, (c) depending on your relative performance (i.e., how your work performance compares to that of your peers), you would be working in a **non-unionized** environment; and, (d) there would be approximately a **1:84** staff to patient ratio.

**These jobs are otherwise identical in all other aspects.**

Now consider the situation where you are given the jobs offered above when you are aged 30, and you have decided to accept one of these jobs. What is the percent (%) chance (or chances out of 100) that you will choose each of these jobs?

The chance of each job should be a number between 0 and 100 and the chances given to the three jobs should add up to 100.

**Percent Chance**

Job 1 _____________

Job 2 _____________

Job 3 _____________

1. You have been offered the following 3 jobs when you are aged 30.

|  | (a) | (b) | (c) | (d) |
| --- | --- | --- | --- | --- |
|  | Earnings per year working full-time | Probability of you being fired or laid off from the job in the next year | Amount of bonus based on relative performance (% of income) | Proportion of men in the firm in similar positions |
| Job 1 | $39.29 | 4% | Union | 1:85 |
| Job 2 | $38.93 | 3% | Non-Union | 1:86 |
| Job 3 | $39.13 | 2% | Union | 1:103 |

For example, if you were to choose Job 1, (a) your annual earnings would be **$39.29** if working full time; (b) the likelihood of being fired from the job over the next year would be **4%**, (c) depending on your relative performance (i.e., how your work performance compares to that of your peers), you would be working in a **Unionized** environment; and, (d) there would be approximately a **1:85** staff to patient ratio.

**These jobs are otherwise identical in all other aspects.**

Now consider the situation where you are given the jobs offered above when you are aged 30, and you have decided to accept one of these jobs. What is the percent (%) chance (or chances out of 100) that you will choose each of these jobs?

The chance of each job should be a number between 0 and 100 and the chances given to the three jobs should add up to 100.

**Percent Chance**

Job 1 _____________

Job 2 _____________

Job 3 _____________

1. You have been offered the following 3 jobs when you are aged 30.

|  | (a) | (b) | (c) | (d) |
| --- | --- | --- | --- | --- |
|  | Earnings per year working full-time | Probability of you being fired or laid off from the job in the next year | Amount of bonus based on relative performance (% of income) | Proportion of men in the firm in similar positions |
| Job 1 | $34.58 | 6% | Non-Union | 1:72 |
| Job 2 | $39.51 | 7% | Union | 1:117 |
| Job 3 | $35.34 | 2% | Non-Union | 1:82 |

For example, if you were to choose Job 1, (a) your annual earnings would be **$34.58** if working full time; (b) the likelihood of being fired from the job over the next year would be **6%**, (c) depending on your relative performance (i.e., how your work performance compares to that of your peers), you would be working in a **non-unionized** environment; and, (d) there would be approximately a **1:72** staff to patient ratio.

**These jobs are otherwise identical in all other aspects.**

Now consider the situation where you are given the jobs offered above when you are aged 30, and you have decided to accept one of these jobs. What is the percent (%) chance (or chances out of 100) that you will choose each of these jobs?

The chance of each job should be a number between 0 and 100 and the chances given to the three jobs should add up to 100.

**Percent Chance**

Job 1 _____________

Job 2 _____________

Job 3 _____________

1. You have been offered the following 3 jobs when you are aged 30.

|  | (a) | (b) | (c) | (d) |
| --- | --- | --- | --- | --- |
|  | Earnings per year working full-time | Probability of you being fired or laid off from the job in the next year | Amount of bonus based on relative performance (% of income) | Proportion of men in the firm in similar positions |
| Job 1 | $38.75 | 7% | Union | 1:114 |
| Job 2 | $36.88 | 1% | Non-Union | 1:53 |
| Job 3 | $37.69 | 3% | Union | 1:99 |

For example, if you were to choose Job 1, (a) your annual earnings would be **$38.75** if working full time; (b) the likelihood of being fired from the job over the next year would be **7%**, (c) depending on your relative performance (i.e., how your work performance compares to that of your peers), you would be working in a **Unionized** environment; and, (d) there would be approximately a **1:114** staff to patient ratio.

**These jobs are otherwise identical in all other aspects.**

Now consider the situation where you are given the jobs offered above when you are aged 30, and you have decided to accept one of these jobs. What is the percent (%) chance (or chances out of 100) that you will choose each of these jobs?

The chance of each job should be a number between 0 and 100 and the chances given to the three jobs should add up to 100.

**Percent Chance**

Job 1 _____________

Job 2 _____________

Job 3 _____________

1. You have been offered the following 3 jobs when you are aged 30.

|  | (a) | (b) | (c) | (d) |
| --- | --- | --- | --- | --- |
|  | Earnings per year working full-time | Probability of you being fired or laid off from the job in the next year | Amount of bonus based on relative performance (% of income) | Proportion of men in the firm in similar positions |
| Job 1 | $35.11 | 3% | Non-Union | 1:87 |
| Job 2 | $41.18 | 5% | Union | 1:111 |
| Job 3 | $36.43 | 6% | Non-Union | 1:56 |

For example, if you were to choose Job 1, (a) your annual earnings would be **$35.11** if working full time; (b) the likelihood of being fired from the job over the next year would be **3%**, (c) depending on your relative performance (i.e., how your work performance compares to that of your peers), you would be working in a **non-unionized** environment; and, (d) there would be approximately a **1:87** staff to patient ratio.

**These jobs are otherwise identical in all other aspects.**

Now consider the situation where you are given the jobs offered above when you are aged 30, and you have decided to accept one of these jobs. What is the percent (%) chance (or chances out of 100) that you will choose each of these jobs?

The chance of each job should be a number between 0 and 100 and the chances given to the three jobs should add up to 100.

**Percent Chance**

Job 1 _____________

Job 2 _____________

Job 3 _____________

1. You have been offered the following 3 jobs when you are aged 30.

|  | (a) | (b) | (c) | (d) |
| --- | --- | --- | --- | --- |
|  | Earnings per year working full-time | Probability of you being fired or laid off from the job in the next year | Amount of bonus based on relative performance (% of income) | Proportion of men in the firm in similar positions |
| Job 1 | $37.77 | 1% | Union | 1:98 |
| Job 2 | $40.59 | 4% | Non-Union | 1:102 |
| Job 3 | $37.44 | 2% | Union | 1:57 |

For example, if you were to choose Job 1, (a) your annual earnings would be **$37.77** if working full time; (b) the likelihood of being fired from the job over the next year would be **1%**, (c) depending on your relative performance (i.e., how your work performance compares to that of your peers), you would be working in a **Unionized** environment; and, (d) there would be approximately a **1:98** staff to patient ratio.

**These jobs are otherwise identical in all other aspects.**

Now consider the situation where you are given the jobs offered above when you are aged 30, and you have decided to accept one of these jobs. What is the percent (%) chance (or chances out of 100) that you will choose each of these jobs?

The chance of each job should be a number between 0 and 100 and the chances given to the three jobs should add up to 100.

**Percent Chance**

Job 1 _____________

Job 2 _____________

Job 3 _____________

1. You have been offered the following 3 jobs when you are aged 30.

|  | (a) | (b) | (c) | (d) |
| --- | --- | --- | --- | --- |
|  | Earnings per year working full-time | Probability of you being fired or laid off from the job in the next year | Amount of bonus based on relative performance (% of income) | Proportion of men in the firm in similar positions |
| Job 1 | $34.44 | 3% | Non-Union | 1:106 |
| Job 2 | $36.55 | 7% | Union | 1:113 |
| Job 3 | $34.33 | 5% | Non-Union | 1:97 |

For example, if you were to choose Job 1, (a) your annual earnings would be **$34.44** if working full time; (b) the likelihood of being fired from the job over the next year would be **3%**, (c) depending on your relative performance (i.e., how your work performance compares to that of your peers),you would be working in a **non-unionized** environment; and, (d) there would be approximately a **1:106** staff to patient ratio.

**These jobs are otherwise identical in all other aspects.**

Now consider the situation where you are given the jobs offered above when you are aged 30, and you have decided to accept one of these jobs. What is the percent (%) chance (or chances out of 100) that you will choose each of these jobs?

The chance of each job should be a number between 0 and 100 and the chances given to the three jobs should add up to 100.

**Percent Chance**

Job 1 _____________

Job 2 _____________

Job 3 _____________

## Job Offer Scenarios: Section C

In each of the scenarios in this section, you will be shown hypothetical job offers. Each job offer is characterized by:

- Hourly wage earnings working full-time
- Amount of vacation or paid time off
- Shift work/rotation/preferable hours (evening, morning, afternoon)
- Opportunity for training and development

These jobs are otherwise identical in all other aspects.

When answering these questions, envision that you have completed your program, graduated and are seeking employment in the nursing field. You have been offered each of these jobs, and now you have to decide which one to choose.

In each scenario, you will be asked for the percent chance (or chances out of 100) of the likelihood of you choosing each of the alternatives. State each chance as a number between 0 and 100 and the chances given to the three alternatives should **add up to 100**.

- 2-5% would mean “almost no chance”
- ~19% would be along the lines of “not much chance”
- 47-55% would mean “pretty even chance”
- ~82% would indicate there to be “a very good chance”
- 95-98% would mean “almost certain”

For example, if you give 3% chance to one option, it means that there is a very low possibility that you would choose that job scenario. On the other hand, if you give an 88% chance to a job scenario, it means that it is quite likely that you will choose it.

1. You have been offered the following 3 jobs when you are aged 30.

|  | (a) | (b) | (c) | (d) |
| --- | --- | --- | --- | --- |
|  | Earnings per year working full-time | Amount of vacation or paid time off | Shift work/ rotation/ preferable hours | Opportunity for training and development |
| Job 1 | $36.54 | 4 weeks | Consistent Shifts | $2208 |
| Job 2 | $39.00 | 3 weeks | Rotation Shifts | $3996 |
| Job 3 | $40.93 | 2 weeks | Consistent Shifts | $3956 |

For example, if you were to choose Job 1, (a) your annual earnings would be **$36.54** if working full time; (b) You would have **4 weeks** of paid time off work per calendar year, (c) Your shift rotation would be **consistent** days/nights/evenings; and, (d) There is **$2,208** funds available for training and development.

**These jobs are otherwise identical in all other aspects.**

Now consider the situation where you are given the jobs offered above when you are aged 30, and you have decided to accept one of these jobs. What is the percent (%) chance (or chances out of 100) that you will choose each of these jobs?

The chance of each job should be a number between 0 and 100 and the chances given to the three jobs should add up to 100.

**Percent Chance**

Job 1 _____________

Job 2 _____________

Job 3 _____________

1. You have been offered the following 3 jobs when you are aged 30.

|  | (a) | (b) | (c) | (d) |
| --- | --- | --- | --- | --- |
|  | Earnings per year working full-time | Amount of vacation or paid time off | Shift work/ rotation/ preferable hours | Opportunity for training and development |
| Job 1 | $38.44 | 3 weeks | Rotation Shifts | $3294 |
| Job 2 | $34.54 | 4 weeks | Consistent Shifts | $2507 |
| Job 3 | $39.41 | 2 weeks | Rotation Shifts | $3562 |

For example, if you were to choose Job 1, (a) your annual earnings would be **$38.44** if working full time; (b) You would have **3 weeks** of paid time off work per calendar year, (c) Your shift rotation would be **Rotation** days/nights/evenings; and, (d) There is **$3,294** funds available for training and development.

**These jobs are otherwise identical in all other aspects.**

Now consider the situation where you are given the jobs offered above when you are aged 30, and you have decided to accept one of these jobs. What is the percent (%) chance (or chances out of 100) that you will choose each of these jobs?

The chance of each job should be a number between 0 and 100 and the chances given to the three jobs should add up to 100.

**Percent Chance**

Job 1 _____________

Job 2 _____________

Job 3 _____________

1. You have been offered the following 3 jobs when you are aged 30.

|  | (a) | (b) | (c) | (d) |
| --- | --- | --- | --- | --- |
|  | Earnings per year working full-time | Amount of vacation or paid time off | Shift work/ rotation/ preferable hours | Opportunity for training and development |
| Job 1 | $37.80 | 3 weeks | Consistent Shifts | $4075 |
| Job 2 | $33.80 | 4 weeks | Rotation Shifts | $5797 |
| Job 3 | $39.69 | 2 weeks | Consistent Shifts | $5220 |

For example, if you were to choose Job 1, (a) your annual earnings would be **$37.80** if working full time; (b) You would have **3 weeks** of paid time off work per calendar year, (c) Your shift rotation would be **consistent** days/nights/evenings; and, (d) There is **$4,075** funds available for training and development.

**These jobs are otherwise identical in all other aspects.**

Now consider the situation where you are given the jobs offered above when you are aged 30, and you have decided to accept one of these jobs. What is the percent (%) chance (or chances out of 100) that you will choose each of these jobs?

The chance of each job should be a number between 0 and 100 and the chances given to the three jobs should add up to 100.

**Percent Chance**

Job 1 _____________

Job 2 _____________

Job 3 _____________

1. You have been offered the following 3 jobs when you are aged 30.

|  | (a) | (b) | (c) | (d) |
| --- | --- | --- | --- | --- |
|  | Earnings per year working full-time | Amount of vacation or paid time off | Shift work/ rotation/ preferable hours | Opportunity for training and development |
| Job 1 | $35.26 | 3 weeks | Rotation Shifts | $3109 |
| Job 2 | $34.18 | 4 weeks | Consistent Shifts | $5530 |
| Job 3 | $36.48 | 2 weeks | Rotation Shifts | $3495 |

For example, if you were to choose Job 1, (a) your annual earnings would be **$35.26** if working full time; (b) You would have **3 weeks** of paid time off work per calendar year, (c) Your shift rotation would be **rotation** days/nights/evenings; and, (d) There is **$3,109** funds available for training and development.

**These jobs are otherwise identical in all other aspects.**

Now consider the situation where you are given the jobs offered above when you are aged 30, and you have decided to accept one of these jobs. What is the percent (%) chance (or chances out of 100) that you will choose each of these jobs?

The chance of each job should be a number between 0 and 100 and the chances given to the three jobs should add up to 100.

**Percent Chance**

Job 1 _____________

Job 2 _____________

Job 3 _____________

1. You have been offered the following 3 jobs when you are aged 30.

|  | (a) | (b) | (c) | (d) |
| --- | --- | --- | --- | --- |
|  | Earnings per year working full-time | Amount of vacation or paid time off | Shift work/ rotation/ preferable hours | Opportunity for training and development |
| Job 1 | $37.81 | 3 weeks | Consistent Shifts | $3853 |
| Job 2 | $36.37 | 4 weeks | Rotation Shifts | $5142 |
| Job 3 | $37.88 | 2 weeks | Consistent Shifts | $4433 |

For example, if you were to choose Job 1, (a) your annual earnings would be **$37.81** if working full time; (b) You would have **3 weeks** of paid time off work per calendar year, (c) Your shift rotation would be **consistent** days/nights/evenings; and, (d) There is **$3,853** funds available for training and development.

**These jobs are otherwise identical in all other aspects.**

Now consider the situation where you are given the jobs offered above when you are aged 30, and you have decided to accept one of these jobs. What is the percent (%) chance (or chances out of 100) that you will choose each of these jobs?

The chance of each job should be a number between 0 and 100 and the chances given to the three jobs should add up to 100.

**Percent Chance**

Job 1 _____________

Job 2 _____________

Job 3 _____________

1. You have been offered the following 3 jobs when you are aged 30.

|  | (a) | (b) | (c) | (d) |
| --- | --- | --- | --- | --- |
|  | Earnings per year working full-time | Amount of vacation or paid time off | Shift work/ rotation/ preferable hours | Opportunity for training and development |
| Job 1 | $40.48 | 2 weeks | Rotation Shifts | $3939 |
| Job 2 | $34.03 | 4 weeks | Consistent Shifts | $5037 |
| Job 3 | $39.42 | 3 weeks | Rotation Shifts | $2559 |

For example, if you were to choose Job 1, (a) your annual earnings would be **$40.48** if working full time; (b) You would have **2 weeks** of paid time off work per calendar year, (c) Your shift rotation would be **rotation** days/nights/evenings; and, (d) There is **$3,939** funds available for training and development.

**These jobs are otherwise identical in all other aspects.**

Now consider the situation where you are given the jobs offered above when you are aged 30, and you have decided to accept one of these jobs. What is the percent (%) chance (or chances out of 100) that you will choose each of these jobs?

The chance of each job should be a number between 0 and 100 and the chances given to the three jobs should add up to 100.

**Percent Chance**

Job 1 _____________

Job 2 _____________

Job 3 _____________

1. You have been offered the following 3 jobs when you are aged 30.

|  | (a) | (b) | (c) | (d) |
| --- | --- | --- | --- | --- |
|  | Earnings per year working full-time | Amount of vacation or paid time off | Shift work/ rotation/ preferable hours | Opportunity for training and development |
| Job 1 | $41.52 | 3 weeks | Consistent Shifts | $4605 |
| Job 2 | $38.21 | 2 weeks | Rotation Shifts | $3317 |
| Job 3 | $39.84 | 4 weeks | Consistent Shifts | $3986 |

For example, if you were to choose Job 1, (a) your annual earnings would be **$41.52** if working full time; (b) You would have **3 weeks** of paid time off work per calendar year, (c) Your shift rotation would be **consistent** days/nights/evenings; and, (d) There is **$4,605** funds available for training and development.

**These jobs are otherwise identical in all other aspects.**

Now consider the situation where you are given the jobs offered above when you are aged 30, and you have decided to accept one of these jobs. What is the percent (%) chance (or chances out of 100) that you will choose each of these jobs?

The chance of each job should be a number between 0 and 100 and the chances given to the three jobs should add up to 100.

**Percent Chance**

Job 1 _____________

Job 2 _____________

Job 3 _____________

1. You have been offered the following 3 jobs when you are aged 30.

|  | (a) | (b) | (c) | (d) |
| --- | --- | --- | --- | --- |
|  | Earnings per year working full-time | Amount of vacation or paid time off | Shift work/ rotation/ preferable hours | Opportunity for training and development |
| Job 1 | $36.17 | 4 weeks | Rotation Shifts | $4640 |
| Job 2 | $39.25 | 2 weeks | Consistent Shifts | $5146 |
| Job 3 | $37.13 | 3 weeks | Rotation Shifts | $5912 |

For example, if you were to choose Job 1, (a) your annual earnings would be **$36.17** if working full time; (b) You would have **4 weeks** of paid time off work per calendar year, (c) Your shift rotation would be **rotation** days/nights/evenings; and, (d) There is **$4,640** funds available for training and development.

**These jobs are otherwise identical in all other aspects.**

Now consider the situation where you are given the jobs offered above when you are aged 30, and you have decided to accept one of these jobs. What is the percent (%) chance (or chances out of 100) that you will choose each of these jobs?

The chance of each job should be a number between 0 and 100 and the chances given to the three jobs should add up to 100.

**Percent Chance**

Job 1 _____________

Job 2 _____________

Job 3 _____________

## Job Offer Scenarios: Section D

In each of the scenarios in this section, you will be shown hypothetical job offers. Each job offer is characterized by:

- Earnings per year working full-time
- Available benefits and pension package
- Risk of injury (physical, verbal and psychosocial trauma)
- Patient acuity (how complex are the patients?)

These jobs are otherwise identical in all other aspects.

When answering these questions, envision that you have completed your program, graduated and are seeking employment in the nursing field. You have been offered each of these jobs, and now you have to decide which one to choose.

In each scenario, you will be asked for the percent chance (or chances out of 100) of the likelihood of you choosing each of the alternatives. State each chance as a number between 0 and 100 and the chances given to the three alternatives should **add up to 100**.

- 2-5% would mean “almost no chance”
- ~19% would be along the lines of “not much chance”
- 47-55% would mean “pretty even chance”
- ~82% would indicate there to be “a very good chance”
- 95-98% would mean “almost certain”

For example, if you give 3% chance to one option, it means that there is a very low possibility that you would choose that job scenario. On the other hand, if you give an 88% chance to a job scenario, it means that it is quite likely that you will choose it.

1. You have been offered the following 3 jobs when you are aged 30.

|  | (a) | (b) | (c) | (d) |
| --- | --- | --- | --- | --- |
|  | Earnings per year working full-time | Available benefits and pension package | Risk of injury on the job | Patient acuity |
| Job 1 | $36.84 | 13% | 6.2 | Stable |
| Job 2 | $36.67 | 10% | 13.7 | Moderate |
| Job 3 | $40.63 | 9% | 24.8 | Complex |

For example, if you were to choose Job 1, (a) your annual earnings would be **$36.84** if working full time; (b) **13%** available for benefits/pension, (c) The likelihood of you being injured on the job would be **6.2** out of 100; and, (d) patients that you would be providing care for are **stable**.

**These jobs are otherwise identical in all other aspects.**

Now consider the situation where you are given the jobs offered above when you are aged 30, and you have decided to accept one of these jobs. What is the percent (%) chance (or chances out of 100) that you will choose each of these jobs?

The chance of each job should be a number between 0 and 100 and the chances given to the three jobs should add up to 100.

**Percent Chance**

Job 1 _____________

Job 2 _____________

Job 3 _____________

1. You have been offered the following 3 jobs when you are aged 30.

|  | (a) | (b) | (c) | (d) |
| --- | --- | --- | --- | --- |
|  | Earnings per year working full-time | Available benefits and pension package | Risk of injury on the job | Patient acuity |
| Job 1 | $34.19 | 12% | 16.9 | High Risk |
| Job 2 | $39.45 | 9% | 28.0 | Stable |
| Job 3 | $34.03 | 11% | 13.1 | Moderate |

For example, if you were to choose Job 1, (a) your annual earnings would be **$34.19** if working full time; (b) **12%** available for benefits/pension, (c) The likelihood of you being injured on the job would be **16.9** out of 100; and, (d) patients that you would be providing care for are **high risk**.

**These jobs are otherwise identical in all other aspects.**

Now consider the situation where you are given the jobs offered above when you are aged 30, and you have decided to accept one of these jobs. What is the percent (%) chance (or chances out of 100) that you will choose each of these jobs?

The chance of each job should be a number between 0 and 100 and the chances given to the three jobs should add up to 100.

**Percent Chance**

Job 1 _____________

Job 2 _____________

Job 3 _____________

1. You have been offered the following 3 jobs when you are aged 30.

|  | (a) | (b) | (c) | (d) |
| --- | --- | --- | --- | --- |
|  | Earnings per year working full-time | Available benefits and pension package | Risk of injury on the job | Patient acuity |
| Job 1 | $36.95 | 10% | 25.3 | Complex |
| Job 2 | $36.39 | 13% | 12.1 | High Risk |
| Job 3 | $37.84 | 10% | 7.4 | Stable |

For example, if you were to choose Job 1, (a) your annual earnings would be **$36.95** if working full time; (b) **10%** available for benefits/pension, (c) The likelihood of you being injured on the job would be **25.3** out of 100; and, (d) patients that you would be providing care for are **complex**.

**These jobs are otherwise identical in all other aspects.**

Now consider the situation where you are given the jobs offered above when you are aged 30, and you have decided to accept one of these jobs. What is the percent (%) chance (or chances out of 100) that you will choose each of these jobs?

The chance of each job should be a number between 0 and 100 and the chances given to the three jobs should add up to 100.

**Percent Chance**

Job 1 _____________

Job 2 _____________

Job 3 _____________

1. You have been offered the following 3 jobs when you are aged 30.

|  | (a) | (b) | (c) | (d) |
| --- | --- | --- | --- | --- |
|  | Earnings per year working full-time | Available benefits and pension package | Risk of injury on the job | Patient acuity |
| Job 1 | $37.38 | 13% | 19.5 | Moderate |
| Job 2 | $39.50 | 10% | 27.6 | Complex |
| Job 3 | $39.26 | 11% | 8.3 | High Risk |

For example, if you were to choose Job 1, (a) your annual earnings would be **$37.38** if working full time; (b) **12 %** available for benefits/pension, (c) The likelihood of you being injured on the job would be **19.5** out of 100; and, (d) patients that you would be providing care for are **moderate** complexity.

**These jobs are otherwise identical in all other aspects.**

Now consider the situation where you are given the jobs offered above when you are aged 30, and you have decided to accept one of these jobs. What is the percent (%) chance (or chances out of 100) that you will choose each of these jobs?

The chance of each job should be a number between 0 and 100 and the chances given to the three jobs should add up to 100.

**Percent Chance**

Job 1 _____________

Job 2 _____________

Job 3 _____________

1. You have been offered the following 3 jobs when you are aged 30.

|  | (a) | (b) | (c) | (d) |
| --- | --- | --- | --- | --- |
|  | Earnings per year working full-time | Available benefits and pension package | Risk of injury on the job | Patient acuity |
| Job 1 | $35.99 | 12% | 26.9 | Stable |
| Job 2 | $36.78 | 9% | 6.4 | Moderate |
| Job 3 | $34.35 | 11% | 15.0 | Complex |

For example, if you were to choose Job 1, (a) your annual earnings would be **$35.99** if working full time; (b) **9 %** available for benefits/pension, (c) The likelihood of you being injured on the job would be **26.9** out of 100; and, (d) patients that you would be providing care for are **stable**.

**These jobs are otherwise identical in all other aspects.**

Now consider the situation where you are given the jobs offered above when you are aged 30, and you have decided to accept one of these jobs. What is the percent (%) chance (or chances out of 100) that you will choose each of these jobs?

The chance of each job should be a number between 0 and 100 and the chances given to the three jobs should add up to 100.

**Percent Chance**

Job 1 _____________

Job 2 _____________

Job 3 _____________

1. You have been offered the following 3 jobs when you are aged 30.

|  | (a) | (b) | (c) | (d) |
| --- | --- | --- | --- | --- |
|  | Earnings per year working full-time | Available benefits and pension package | Risk of injury on the job | Patient acuity |
| Job 1 | $41.71 | 9% | 12.7 | High Risk |
| Job 2 | $35.47 | 13% | 18.3 | Stable |
| Job 3 | $38.03 | 11% | 22.6 | Moderate |

For example, if you were to choose Job 1, (a) your annual earnings would be **$41.71** if working full time; (b) **9 %** available for benefits/pension, (c) The likelihood of you being injured on the job would be **12.7** out of 100; and, (d) patients that you would be providing care for are **high risk**.

**These jobs are otherwise identical in all other aspects.**

Now consider the situation where you are given the jobs offered above when you are aged 30, and you have decided to accept one of these jobs. What is the percent (%) chance (or chances out of 100) that you will choose each of these jobs?

The chance of each job should be a number between 0 and 100 and the chances given to the three jobs should add up to 100.

**Percent Chance**

Job 1 _____________

Job 2 _____________

Job 3 _____________

1. You have been offered the following 3 jobs when you are aged 30.

|  | (a) | (b) | (c) | (d) |
| --- | --- | --- | --- | --- |
|  | Earnings per year working full-time | Available benefits and pension package | Risk of injury on the job | Patient acuity |
| Job 1 | $35.94 | 12% | 7.1 | Complex |
| Job 2 | $36.18 | 10% | 27.8 | High Risk |
| Job 3 | $36.38 | 9% | 16.4 | Stable |

For example, if you were to choose Job 1, (a) your annual earnings would be **$35.94** if working full time; (b) **12 %** available for benefits/pension, (c) The likelihood of you being injured on the job would be **7.1** out of 100; and, (d) patients that you would be providing care for are **complex**.

**These jobs are otherwise identical in all other aspects.**

Now consider the situation where you are given the jobs offered above when you are aged 30, and you have decided to accept one of these jobs. What is the percent (%) chance (or chances out of 100) that you will choose each of these jobs?

The chance of each job should be a number between 0 and 100 and the chances given to the three jobs should add up to 100.

**Percent Chance**

Job 1 _____________

Job 2 _____________

Job 3 _____________

1. You have been offered the following 3 jobs when you are aged 30.

|  | (a) | (b) | (c) | (d) |
| --- | --- | --- | --- | --- |
|  | Earnings per year working full-time | Available benefits and pension package | Risk of injury on the job | Patient acuity |
| Job 1 | $37.80 | 11% | 8.5 | Moderate |
| Job 2 | $41.97 | 9% | 27.2 | Complex |
| Job 3 | $35.99 | 13% | 13.4 | High Risk |

For example, if you were to choose Job 1, (a) your annual earnings would be **$37.80** if working full time; (b) **11 %** available for benefits/pension, (c) The likelihood of you being injured on the job would be **8.5** out of 100; and, (d) patients that you would be providing care for are **moderate** complexity.

**These jobs are otherwise identical in all other aspects.**

Now consider the situation where you are given the jobs offered above when you are aged 30, and you have decided to accept one of these jobs. What is the percent (%) chance (or chances out of 100) that you will choose each of these jobs?

The chance of each job should be a number between 0 and 100 and the chances given to the three jobs should add up to 100.

**Percent Chance**

Job 1 _____________

Job 2 _____________

Job 3 _____________

Are you interested in participating in a focus group discussing work attributes that you would find attractive to work in the continuing care sector? Y/N

(conditional on above response) Please provide your contact information (email): ___________________

Would you like to receive a summary of the results from the study? Y/N

(conditional on above response) Please provide your contact information (email): ___________________

If you would like to be added to the draw for 1 of 4 $250 gift cards, please enter your email (only winners will be contacted): _________________

| A | Hourly Wage | % increase per year | Hours of work | Part time available |
| --- | --- | --- | --- | --- |
| 1 | $35.36 | 2% | 35 | Yes |
| 2 | $35.94 | 2.5% | 40 | No |
| 3 | $34.00 | 3% | 44 | Yes |
| 1 | $40.48 | 3% | 37.5 | No |
| 2 | $41.65 | 2% | 40 | Yes |
| 3 | $35.88 | 4% | 35 | No |
| 1 | $40.56 | 2.5% | 44 | Yes |
| 2 | $36.09 | 5% | 40 | No |
| 3 | $40.41 | 3% | 37.5 | Yes |
| 1 | $33.75 | 4% | 44 | No |
| 2 | $41.30 | 2% | 50 | Yes |
| 3 | $38.67 | 3% | 40 | No |
| 1 | $38.10 | 3% | 48 | Yes |
| 2 | $34.17 | 4.5% | 35 | No |
| 3 | $40.49 | 2.5% | 40 | Yes |
| 1 | $35.31 | 2% | 37.5 | No |
| 2 | $33.88 | 4% | 44 | Yes |
| 3 | $35.00 | 3% | 48 | No |
| 1 | $39.92 | 2% | 35 | Yes |
| 2 | $35.41 | 5% | 44 | No |
| 3 | $38.17 | 3% | 40 | Yes |
| 1 | $34.05 | 3% | 40 | No |
| 2 | $34.06 | 2.5% | 44 | Yes |
| 3 | $36.00 | 2% | 50 | No |
| B | Hourly Wage | Probability of being laid off in the next year | Union or Non-Union | Staff-to-patient ratio |
| 1 | $38.97 | 1% | Union | 1: 61 |
| 2 | $38.33 | 3% | Non-Union | 1: 90 |
| 3 | $38.48 | 7% | Union | 1: 56 |
| 1 | $39.52 | 5% | Non-Union | 1: 84 |
| 2 | $40.62 | 6% | Union | 1: 114 |
| 3 | $39.05 | 2% | Non-Union | 1: 69 |
| 1 | $39.29 | 4% | Union | 1: 85 |
| 2 | $38.93 | 3% | Non-Union | 1: 86 |
| 3 | $39.13 | 2% | Union | 1: 103 |
| 1 | $34.58 | 6% | Non-Union | 1: 72 |
| 2 | $39.51 | 7% | Union | 1: 117 |
| 3 | $35.34 | 2% | Non-Union | 1: 82 |
| 1 | $38.75 | 7% | Union | 1: 114 |
| 2 | $36.88 | 1% | Non-Union | 1: 53 |
| 3 | $37.69 | 3% | Union | 1: 99 |
| 1 | $35.11 | 3% | Non-Union | 1: 87 |
| 2 | $41.18 | 5% | Union | 1: 111 |
| 3 | $36.43 | 6% | Non-Union | 1: 56 |
| 1 | $37.77 | 1% | Union | 1: 98 |
| 2 | $40.59 | 4% | Non-Union | 1: 102 |
| 3 | $37.44 | 2% | Union | 1: 57 |
| 1 | $34.44 | 3% | Non-Union | 1: 106 |
| 2 | $36.55 | 7% | Union | 1: 113 |
| 3 | $34.33 | 5% | Non-Union | 1: 97 |
| C | Hourly Wage | Vacation/paid time off | Shift/preferable hours | Annual allowance for training and development |
| 1 | $36.54 | 4 weeks | Consistent Shifts | $2208 |
| 2 | $39.00 | 3 weeks | Rotation Shifts | $3996 |
| 3 | $40.93 | 2 weeks | Consistent Shifts | $3956 |
| 1 | $38.44 | 3 weeks | Rotation Shifts | $3294 |
| 2 | $34.54 | 4 weeks | Consistent Shifts | $2507 |
| 3 | $39.41 | 2 weeks | Rotation Shifts | $3562 |
| 1 | $37.80 | 3 weeks | Consistent Shifts | $4075 |
| 2 | $33.80 | 4 weeks | Rotation Shifts | $5797 |
| 3 | $39.69 | 2 weeks | Consistent Shifts | $5220 |
| 1 | $35.26 | 3 weeks | Rotation Shifts | $3109 |
| 2 | $34.18 | 4 weeks | Consistent Shifts | $5530 |
| 3 | $36.48 | 2 weeks | Rotation Shifts | $3495 |
| 1 | $37.81 | 3 weeks | Consistent Shifts | $3853 |
| 2 | $36.37 | 4 weeks | Rotation Shifts | $5142 |
| 3 | $37.88 | 2 weeks | Consistent Shifts | $4433 |
| 1 | $40.48 | 2 weeks | Rotation Shifts | $3939 |
| 2 | $34.03 | 4 weeks | Consistent Shifts | $5037 |
| 3 | $39.42 | 3 weeks | Rotation Shifts | $2559 |
| 1 | $41.52 | 2 weeks | Consistent Shifts | $4605 |
| 2 | $38.21 | 4 weeks | Rotation Shifts | $3317 |
| 3 | $39.84 | 3 weeks | Consistent Shifts | $3986 |
| 1 | $36.17 | 4 weeks | Rotation Shifts | $4640 |
| 2 | $39.25 | 2 weeks | Consistent Shifts | $5146 |
| 3 | $37.13 | 3 weeks | Rotation Shifts | $5912 |
| D | Hourly Wage | Benefits/pension available | Risk of injury (Rates per 100 FTEs) | Patient acuity |
| 1 | $36.84 | 13% | 6.2 | Stable |
| 2 | $36.67 | 10% | 13.7 | Moderate |
| 3 | $40.63 | 9% | 24.8 | Complex |
| 1 | $34.19 | 12% | 16.9 | High Risk |
| 2 | $39.45 | 9% | 28.0 | Stable |
| 3 | $34.03 | 11% | 13.1 | Moderate |
| 1 | $36.95 | 10% | 25.3 | Complex |
| 2 | $36.39 | 10% | 12.1 | High Risk |
| 3 | $37.84 | 13% | 7.4 | Stable |
| 1 | $37.38 | 12% | 19.5 | Moderate |
| 2 | $39.50 | 10% | 27.6 | Complex |
| 3 | $39.26 | 13% | 8.3 | High Risk |
| 1 | $35.99 | 9% | 26.9 | Stable |
| 2 | $36.78 | 12% | 6.4 | Moderate |
| 3 | $34.35 | 11% | 15.0 | Complex |
| 1 | $41.71 | 9% | 12.7 | High Risk |
| 2 | $35.47 | 13% | 18.3 | Stable |
| 3 | $38.03 | 11% | 22.6 | Moderate |
| 1 | $35.94 | 12% | 7.1 | Complex |
| 2 | $36.18 | 10% | 27.8 | High Risk |
| 3 | $36.38 | 9% | 16.4 | Stable |
| 1 | $37.80 | 11% | 8.5 | Moderate |
| 2 | $41.97 | 9% | 27.2 | Complex |
| 3 | $35.99 | 13% | 13.4 | High Risk |
