# Supplementary material for: Perspectives on Work in the Continuing Care Sector during and after the COVID-19 Pandemic: A Mixed-Method Design
Source: J Nurs Manag. 2024 Apr 18;2024:7187263. doi: 10.1155/2024/7187263 (PMC11919170; doi:10.1155/2024/7187263)
Supplement: Supplementary Materials — The Supplementary Materials that are referenced in the study include: Appendix A: semistructured interview-focus group guide. Appendix B: online cross-sectional survey instrument. Appendix C: job attribute justifications. Appendix D: Table A1 results where the researcher (DR) compared the demographic characteristics of those included in the regression analysis to those who were excluded. [file 7187263.f1.zip › Appendix C - Attribute Justifications.docx]

## Appendix C - Methods for Generating Job Attributes

The survey instrument contained four sections with hypothetical job scenarios. Each section contained eight scenarios, each presenting three job offers. Within each scenario, job offers differed in four attributes. Participants were informed that all other attributes remained the same across the offers. The attributes that were varied included:

- Hourly wage
- Annual percentage increase in earnings
- Average work hours per week for full-time employees
- Work flexibility: Part-time option available
- Probability of being fired or laid off from the job in the next year
- Union or non-unionized work environment
- Staffing ratios
- Amount of vacation or paid time off
- Shift work/rotation/preferable hours
- Opportunity for training and development
- Available benefits and pension package

The values in the job scenarios were randomly selected from realistic ranges. These ranges were determined based on references to the literature, official documentation from the continuing care sector in Ontario, Canada, and expert opinions obtained through consultation with partners in the continuing care sector. We ensured that none of the three job offers were clearly dominant on all attributes by redrawing random values until this condition was met. The following describes how we constructed each of the job attributes used in our scenarios.

- **Hourly wage** – Hourly wage (or income) is an important factor choosing a career path^1^. We decided to use hourly wage over annual salary because nurses generally do shift work and are reimbursed via wages. The registered nurse (RN) Step 1 hourly wage from a local union new hire package^2,3^ in 2017 was listed as $33.70, which we used as the minimum in our range of hourly wages. The research team in consultation with our partners in the continuing care sector agreed upon a range maximum of $41.71. Wages used in the job scenarios were drawn at random from a uniform distribution, with a range of $33.70-$41.71.
- **Annual Percentage increase in earnings** – Annual earnings are an important factor in career choice^1^. We used the percentage range of 2%-5% based on findings of a previous study that concluded their minimum annual increase was 2% and the maximum was 5%^4^. Integer values within this range were randomly selected for each hypothetical job offer.
- **Average work hours per week for full-time** – Hours of work per week ranges depending on where one works. Government funded hospitals and continuing care centers tend to have more controlled hours due to unionized environments, whereas privately funded centers tend to demand greater working hours. It is also not uncommon for nurses to pick up overtime hours. We determined our range of hours worked from two sources: (1) a previous study that found the distribution for hours of work to be average of 47.5 hrs, a minimum of 40 and a maximum of 54^4^; and (2) from a local collective agreement for RN’s stating 37.5 hours per week, and normal hours of work for a nurse are not a guarantee^2^. The research team selected a reasonable hour range of 35-50 hrs per week. Hours worked per week were drawn from a uniform distribution chosen for each hypothetical job scenario.
- **Work Flexibility: Part time option** – Based on the qualitative results from our study, students noted that work flexibility was one of the most important workplace attributes that support recruitment into the continuing care sector. Part-time availability, listed as either ‘yes’ (available) or ‘no’ (not available), was alternated for each hypothetical job scenario.
- **Probability of being fired or laid off from the job in the next year** – Probability of being fired or laid off was listed in the survey as a percentage. The percentages used in our survey were generated from a previous study that found the probability distribution of job security to be 3.88% (mean), with a minimum of 1% and a maximum probability of 7%^4^. We drew integer values from a uniform distribution with a range of 1%-7% to generate the job scenarios.
- **Union vs. Non-union Environment** – Our focus group data revealed that belonging to a unionized work environment was considered a key work attribute for nursing students that would support recruitment to the continuing care sector. The option of unionized environment was alternated for each job scenario, and listed as either Union or Non-Union.
- **Staffing ratios** – This refers to who makes up the care team, which can include: ratios of Registered Nurses to Registered Practical Nurses to Personal Support Workers, on-site physicians, on-site Nurse Practitioners’, and patient to nurse ratios. Depending on the work environment this can be anywhere between 1:50 - 1:120. Input for these numbers were provided by both local regional health departments and Registered Nurses Association of Ontario (RNAO) recommendations^5^. Ratios were drawn from a uniform distribution, within the range of 1:50-1:120 to create the job scenarios.
- **Amount of vacation or paid time off** – According to the local RN collective agreement, full-time RNs with less than 1 year of service receive 1 day per month, up to a maximum of 10 days (2 weeks). Those with less than 5 years of service receive 3 weeks of vacation, while those with 5 years of service receive 4 weeks. Typically, 2 weeks is the minimum vacation duration for entry-level positions. To create realistic scenarios, the research team included a range of 2-4 weeks of vacation, entering either 2, 3, or 4 weeks for each hypothetical job.
- **Shift work/Rotation/Preferable hours –** Shift work is fairly typical for a nursing career across the world^6^. Workplace schedules can vary, including consistent shifts or rotating shifts. In our survey, we considered rotation shifts where employees alternate between day, evening, and night shifts, as well as consistent shifts where employees have a steady schedule of days, evenings, or nights. We evenly alternated both types of shifts when creating hypothetical job scenarios.
- **Opportunity for training and development** - This job attribute refers to the allowance, if any, provided to employees for the purpose of furthering their training and development. The availability of this allowance depends largely on the employer. The range used to generate the job scenarios were based on what the Government of Canada allows for continuing education for nurses (up to $6,000)^7^. The information from the Government web page was accessed during the spring of 2022. For the purpose of the survey, the values for the scenarios were drawn from a uniform distribution with a range of $2,0000 to $6,000.
- **Available benefits and pension package** - Benefits and pension packages are often granted with full time permanent employment. In lieu of benefits, a proportion of salary is used to pay employees based on full time (13%) or part time (9%) employment status. We used this percentage range (9%-13%), extracted from a local region collective agreement^2^, in our survey and randomly generated percentages from a uniform distribution within this range to craft the job scenarios.
- **Risk of injury on the job** - Nurses face several types of injury risks while on the job (physical, verbal, psychosocial trauma, etc). Injury rates were defined as the number of injuries per 100 full-time equivalent (FTE) positions (Rates per 100 FTEs). Based on results from previous literature^8^, values were drawn from a uniform distribution with a range of 6.2-28.3 per 100 FTEs to generate our job scenarios.
- **Patient acuity** - Patient acuity refers to the measurement of intensity of nursing care required by a patient. We used the patient acuity tool^9^ implemented by the American Nursing Association. Each patient is scored on a 1-to-4 scale (1, stable patient; 2, moderate-risk patient; 3, complex patient; 4, high-risk patient) based on the clinical patient characteristics and the care involved (workload). The scale was alternated through each job scenario.

**References**

1. Wiswall M, Zafar B. Determinants of college major choice: Identification using an Information Experiment. *Rev Econ Stud*. 2015;82(2):791-824.

2. Collective Agreement Between: The Regional Municipality of Durham at Lakeview Manor, Beaverton, and Fairview Lodge, Whitby and: Ontario Nurses’ Association. Published online January 2017.

3. Memorandum of Agreement Between: The Regional Municipality of Durham at Lakeview Manor, Beaverton and Fairview Lodge, Whitby and: Ontario Nurses’ Association. Published online April 2018.

4. Wiswall M, Zafar B. Preference for the workplace, investment in human capital, and gender. *Q J Econ*. 2018;133(1):457-507. doi:10.1093/qje/qjx035

5. Nursing Home Basic Care Guarentee: RNAO Submission to the Long-Term Care Staffing Study Advisory Group. Published online June 2020.

6. Dall’Ora C, Dahlgren A. Shift work in nursing: closing the knowledge gaps and advancing innovation in practice. *Int J Nurs Stud*. 2020;112:103743. doi:10.1016/j.ijnurstu.2020.103743

7. Work-life balance, salary and allowances for nurses. Published online September 2022. https://www.sac-isc.gc.ca/eng/1580672006998/1580672137107#sec4

8. Alamgir H, Cvitkovich Y, Yu S, Yassi A. Work-related injury among direct care occupations in British Columbia, Canada. *Occup Environ Med*. 2007;64(11):769-775. doi:10.1136/oem.2006.031914

9. Ingram A, Powell J. Patient acuity tool on medical-surgical unit. Published online April 2018. https://www.myamericannurse.com/patient-acuity-medical-surgical-unit/
