# Supplementary material for: Perspectives on Work in the Continuing Care Sector during and after the COVID-19 Pandemic: A Mixed-Method Design
Source: J Nurs Manag. 2024 Apr 18;2024:7187263. doi: 10.1155/2024/7187263 (PMC11919170; doi:10.1155/2024/7187263)
Supplement: Supplementary Materials — The Supplementary Materials that are referenced in the study include: Appendix A: semistructured interview-focus group guide. Appendix B: online cross-sectional survey instrument. Appendix C: job attribute justifications. Appendix D: Table A1 results where the researcher (DR) compared the demographic characteristics of those included in the regression analysis to those who were excluded. [file 7187263.f1.zip › Appendix D - Table A1.docx]

Table A1. Regression Analysis Demographic Characteristics

|  | **0 (N=51)** | **1 (N=88)** | **Total (N=139)** | **p-value** |
| --- | --- | --- | --- | --- |
| **Year** |  |  |  | 0.377 |
| *1^st^ Year* | 17 (33.3%) | 29 (33.0%) | 46 (33.1%) |  |
| *2^nd^ Year* | 12 (23.5%) | 11 (12.5%) | 23 (16.5%) |  |
| *3^rd^ Year* | 13 (25.5%) | 23 (26.1%) | 36 (25.9%) |  |
| *4^th^ and 5^th^ Year*^1^ | 9 (17.6%) | 25 (28.4%) | 34 (24.4%) |  |
| **Gender** |  |  |  | 0.406 |
| *Male* | 7 (13.7%) | 9 (10.2%) | 16 (11.5%) |  |
| *Female, gender non-conforming, and other*^2^ | 43 (82.4%) | 78 (88.6%) | 120 (86.3%) |  |
| *Prefer not to answer* | 2 (3.9%) | 1 (1.1%) | 3 (2.2%) |  |
| **Age Group** |  |  |  | 0.124 |
| 18-21 | 33 (64.7%) | 39 (44.3%) | 72 (51.8%) |  |
| 22-29 | 11 (21.6%) | 23 (26.1%) | 34 (24.5%) |  |
| 30-39 | 4 (7.8%) | 11 (12.5%) | 15 (10.8%) |  |
| 40-49 | 3 (5.9%) | 11 (12.5%) | 14 (10.1%) |  |
| 50+ | 0 (0.0%) | 4 (4.5%) | 4 (2.9%) |  |
| **Future nursing sector** |  |  |  | 0.472 |
| Home and community care | 15 (29.4%) | 21 (23.9%) | 36 (25.9%) |  |
| Continuing care/Long-term care | 7 (13.7%) | 18 (20.5%) | 25 (18.0%) |  |
| Primary care |  |  |  | 0.818 |
| Primary care | 21 (41.2%) | 38 (43.2%) | 59 (42.4%) |  |
| Acute care | 38 (74.5%) | 67 (76.1%) | 105 (75.5%) |  |
| Other | 0 (0.0%) | 1 (1.1%) | 1 (0.7%) |  |
| **After graduation plans** |  |  |  | 0.437 |
| Work Full-Time | 34 (66.7%) | 69 (78.4%) | 103 (74.1%) |  |
| Work Part-Time | 4 (7.8%) | 5 (5.7%) | 9 (6.5%) |  |
| Attend Graduate/Professional School | 8 (15.7%) | 8 (9.1%) | 16 (11.5%) |  |
| Take time off | 1 (2.0%) | 2 (2.3%) | 3 (2.2%) |  |
| Don't know | 1 (2.0%) | 3 (3.4%) | 4 (2.9%) |  |
| Other | 3 (5.9%) | 1 (1.1%) | 4 (2.9%) |  |
| **Grade point average (GPA)** |  |  |  | 0.375 |
| N-Miss | 5 | 9 | 14 |  |
| Mean (SD) | 3.391 (0.469) | 3.466 (0.441) | 3.438 (0.451) |  |
| Range | 2.0 - 4.2 | 2.2 - 4.2 | 2.0 - 4.2 |  |
| **Salary Expectations** |  |  |  | 0.144 |
| N-Miss | 1 | 5 | 6 |  |
| Mean (SD) | 61940.00 (17010.69) | 67891.57 (25419.89) | 65654.14 (22741.98) |  |
| Range | 9000 - 95000 | 25000 - 250000 | 9000 - 250000 |  |
| ^1^ One respondent in 5^th^ year  ^2^ Combined due to small cell sizes |  |  |  |  |
